# Altered body schema processing in frontotemporal dementia with C9ORF72 mutations

**DOI:** 10.1136/jnnp-2013-306995

**Published:** 2014-02-12

**Authors:** Laura E Downey, Phillip D Fletcher, Hannah L Golden, Colin J Mahoney, Jennifer L Agustus, Jonathan M Schott, Jonathan D Rohrer, Jonathan Beck, Simon Mead, Martin N Rossor, Sebastian J Crutch, Jason D Warren

**Affiliations:** 1 Department of Neurodegenerative Disease, Dementia Research Centre, UCL Institute of Neurology, University College London, London, UK; 2 MRC Prion Unit, Department of Neurodegenerative Disease, UCL Institute of Neurology, University College London, London, UK

**Keywords:** Dementia, Physiology, Neuropsychology, Neuropsychiatry

## Abstract

**Background:**

Mutations in C9ORF72 are an important cause of frontotemporal dementia (FTD) and motor neuron disease. Accumulating evidence suggests that FTD associated with C9ORF72 mutations (C9ORF72-FTD) is distinguished clinically by early prominent neuropsychiatric features that might collectively reflect deranged body schema processing. However, the pathophysiology of C9ORF72-FTD has not been elucidated.

**Methods:**

We undertook a detailed neurophysiological investigation of five patients with C9ORF72-FTD, in relation to patients with FTD occurring sporadically and on the basis of mutations in the microtubule-associated protein tau gene and healthy older individuals. We designed or adapted behavioural tasks systematically to assess aspects of somatosensory body schema processing (tactile discrimination, proprioceptive and body part illusions and self/non-self differentiation).

**Results:**

Patients with C9ORF72-FTD selectively exhibited deficits at these levels of body schema processing in relation to healthy individuals and other patients with FTD.

**Conclusions:**

Altered body schema processing is a novel, generic pathophysiological mechanism that may link the distributed cortico-subcortical network previously implicated in C9ORF72-FTD with a wide range of neuropsychiatric and behavioural symptoms, and constitute a physiological marker of this neurodegenerative proteinopathy.

## Introduction

The frontotemporal lobar degenerations are a heterogeneous group of disorders collectively associated with progressive frontal and temporal lobe atrophy. The most common syndrome, the behavioural variant of frontotemporal dementia (FTD), is characterised by insidious deterioration in behaviour and personality.^1^ In large series, a high proportion of cases have been linked to mutations in either the microtubule-associated binding protein tau gene (MAPT, causing MAPT-FTD); the progranulin gene (GRN, causing GRN-FTD); or expanded hexanucleotide repeat insertions in a non-coding promoter region of open reading frame 72 on chromosome 9 (C9ORF72, causing C9ORF72-FTD). C9ORF72-FTD has recently been identified as a major cause of familial FTD, FTD associated with motor neuron disease and apparently sporadic FTD.^2–4^ Histopathologically, C9ORF72-FTD has been associated with cellular inclusions containing TAR-DNA-binding protein 43 (TDP-43) subtypes A and B and protein p62.^3^ ^5–7^ The pathophysiological mechanisms of C9ORF72-FTD are of particular clinical and neurobiological interest on account of its phenotypic heterogeneity and certain specific phenotypic features. Approximately 40–60% of cases across series have had early, salient neuropsychiatric disturbances,^4^ ^8^ ^9^ including anxiety, agitation and psychotic symptoms of hallucinations and delusions in a substantial though variable proportion (up to around 40% of cases).^3^ ^4^ ^10–12^ Hallucinations and delusions in C9ORF72-FTD are phenomenologically similar to those of schizophrenia, but often have a somatic focus or include prominent elements of disordered awareness of self in relation to others, including themes of paranoia, infestation, bodily distortion or invasion, pregnancy, or loss of voluntary or sphincteric muscle control.^3^ ^4^ ^9^ ^12^ A distributed profile of brain atrophy has been identified in group neuroimaging studies of C9ORF72-FTD, particularly involving frontal and parietal lobes, thalamus and cerebellum.^3^ ^6^ ^13^ Cerebellar atrophy is a longitudinal signal of advancing disease,^14^ and the cerebellum is also a key locus of tissue pathology in C9ORF72-FTD.^3^ ^5^ ^7^ This neuroanatomical evidence suggests involvement of a cortico-thalamo cerebellar network may play an important role in the pathogenesis of C9ORF72-FTD.

Neuropsychiatric features of C9ORF72-FTD might be interpreted mechanistically as arising from aberrant body (or self) schema processing. The concept of ‘body schema’ was first defined by Head and Holmes^15^ as the internalised, combined postural and spatial model of ourselves that provides a standard against which sensory changes can be calibrated and incorporated. The concept has since gained wide currency.^16–22^ Body schema processing and self/non-self differentiation are closely related perceptual and cognitive operations: disambiguation of self from non-self frequently depends on stable and accurate body schema boundaries, modulated by the effects of one's own and external actions. Altered body schema processing has been implicated in the pathogenesis of somatising symptoms,^23^ anxiety,^24^ psychotic disorders and altered states of bodily awareness.^19^ ^25–28^ Disordered processing of sensory information relating to self-image is likely to be of general relevance to a wide range of allied neuropsychiatric phenomena, including some that are not overtly ‘sensory’ (eg, paranoia^29^). We have previously reported the case of a patient with C9ORF72-FTD who was unable reliably to differentiate tactile stimulation arising from his own versus others’ actions.^22^ Furthermore, previous neuroimaging work in healthy individuals and patients with psychosis has implicated a distributed neural network including the cerebellum, parietal lobes, posterior insula and prefrontal cortex in self-referent information processing, particularly ascription of agency to actions.^19^ ^25^ ^30^ ^31^ The behavioural and neuroanatomical features of C9ORF72-FTD overlap substantially with other diseases in the FTD spectrum^3^ ^4^: any disease-associated mechanism is, therefore, unlikely a priori to be the sole mechanism underpinning the phenotype. Nevertheless, the culprit cortico-thalamo cerebellar network implicated in neuroimaging and neuropathological studies of C9ORF72-FTD presents a candidate substrate for altered body schema processing to generate certain neuropsychiatric symptoms exhibited by these patients.^3^ ^22^

Here we investigated systematically physiological and cognitive characteristics of patients with C9ORF72-FTD in relation to healthy older individuals, patients with another genetically mediated FTD syndrome (MAPT-FTD), and patients with sporadic FTD. Our primary objective was to assess body schema processing and the nature and specificity of any disease-associated body schema deficits in C9ORF72-FTD, motivated by previous clinical and neuroanatomical observations in published series. We designed or adapted somatosensory tasks to assess different levels of body schema processing, comprising encoding and modulation of tactile and proprioceptive signals, body part representation and evaluation of the perceptual effects of self versus non-self tactile agency. We hypothesised, first, that patients with C9ORF72-FTD would manifest deficits on these tasks not attributable simply to general cognitive decline; and further, that these deficits would have specificity for C9ORF72-FTD versus other forms of FTD.

## Methods

### Participant details and general assessments

**Figure 1:**
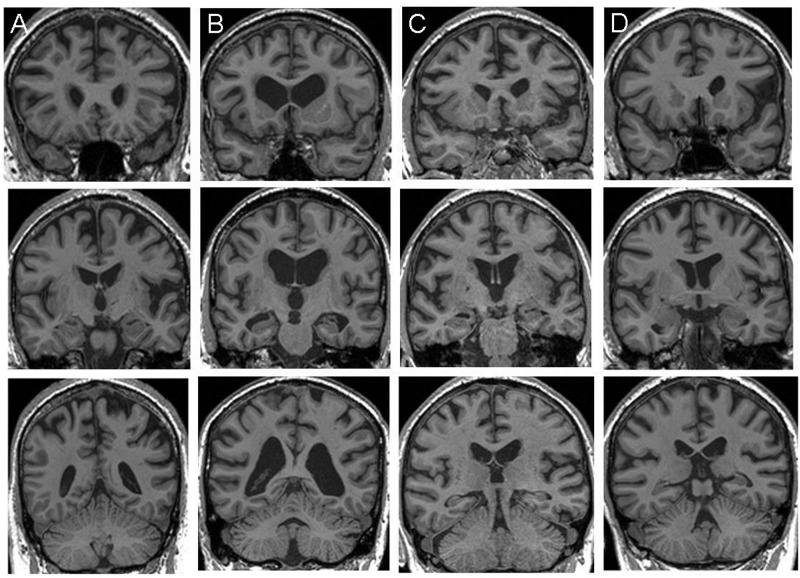
Representative coronal T1-weighted MR brain sections for individual patients (designated A to D) with C9ORF72-associated frontotemporal dementia (MRI was contraindicated in one case). Each column corresponds to a single patient; sections have been selected to capture the anterior frontal lobes and temporal poles (top row), anterior peri-Sylvian regions and medial temporal lobes (middle row), and posterior parietal lobes and cerebellum (bottom row). The left hemisphere is shown on the right in all sections.

Seventeen patients fulfilling current consensus criteria for probable FTD^1^ were recruited from a specialist cognitive disorders clinic, including all patients with genetic FTD who were able to comply with the requirements of the study. Five patients were confirmed to have pathogenic C9ORF72 expansions, seven patients had a pathogenic mutation in the MAPT gene while the remaining five patients had no pathogenic mutations on screening, nor any suggestion of a relevant family history, and were therefore classified as having sporadic FTD (further details of genetic analyses in online supplementary material). Two patients with C9ORF72-FTD had features of early motor neuron disease; no patients had clinical or electrophysiological features of peripheral neuropathy (further details in table 1 and online supplementary material). All patients in the C9ORF72-FTD group exhibited early prominent anxiety, irritability or paranoia; three had somatically focussed preoccupations, one presented with social phobia and one reported auditory hallucinations of voices calling his name. None of the patients had symptoms suggesting a major mood disorder. Structural volumetric brain MRI revealed profiles of brain atrophy in keeping with those previously described in each FTD syndrome^3^ ^32^: patients with C9ORF72-FTD showed variable atrophy profiles including asymmetric selective frontal atrophy, mild fronto-subcortical atrophy, diffuse atrophy and relatively symmetric mesial temporal lobe atrophy (figure 1). No patient had MRI evidence of significant cerebrovascular disease. Cerebrospinal fluid tau:β-amyloid profiles in three patients with C9ORF72-FTD provided no support for concurrent Alzheimer's pathology. Thirteen healthy older individuals with no history of neurological or psychiatric illness also participated in the study. All participants underwent comprehensive clinical and general neuropsychological assessments including the Cambridge Behavioural Inventory completed by the patient's caregiver, and standard tests of general intellectual, executive, social cognition, linguistic, mnestic, semantic, arithmetical and perceptual functions (table 1). Informed consent was obtained for all participants, and the study was approved by the local research ethics committee under Declaration of Helsinki guidelines.

**Table 1:** Demographic, clinical and general neuropsychological characteristics of patient and healthy control groups

	C9ORF72	MAPT	Sporadic FTD	Healthy controls
Clinical features				
Number	5	7	5	13
Age (years)	65 (8)	62 (4)	66 (11)	62 (5)
Sex (M:F)	5:0	5:2	5:0	10:3
Disease duration (years)	7 (3.9)	5 (2.4)	9.3 (6.3)	NA
Handedness (R:L)	5:0	6:0	5:1	13:0
CBI total score (range)	119 (62–168)	128 (57–200)*	104 (30–214)	NA
CBI beliefs score (range)	3.8 (0–12)	2.8 (0–6)*	3.2 (0–7)	NA
Psychiatric symptoms†	5	1	3	NA
Psychotic features	1‡	0	0	NA
*Neuropsychological findings*				
General intellect				
MMSE (/30)	22 (5.4)	24 (5.2)	25 (2.2)	NA
WASI verbal IQ	**83 (22)**	**74 (25)**	**82 (18)**	122 (14)
WASI performance IQ	**90 (25)**	**94 (11)**	**102 (19)**	119 (12)
NART predicted IQ	**103 (19)**	**97 (17)**	**102 (17)**	120 (9)
Episodic memory				
RMT words (/50)	**37 (6.1)**	**32 (5.8)**	**35 (2.5)**	47 (3)
RMT faces (/50)	36 (7.5)	27 (2.8)	**34 (9.0)**	42 (5.9)
Semantic memory				
BPVS (/150)	**132 (17)**	**123 (17)**	**131 (17)**	148 (1.7)
Executive function				
WASI similarities	**26 (6.7)**	**20 (14)**	**22 (14)**	42 (2.9)
WASI matrices	**15 (11)**	**16 (5.7)**	17 (10)	25 (5.1)
D-KEFS Stroop (secs)	**127 (47)**	**87 (43)**	**92 (33)**	52 (11)
Social cognition				
TASIT emotion (/14)	**8.4 (1.6)**	**8.7 (1.6)**	**8.1 (0.6)**§	11 (1.3)¶
TASIT sarcasm (/24)	**15 (4.8)**	**15 (7.7)**	**14 (5.2)**§	22 (2.3)¶
Other skills				
GNT (/30)	20 (3.9)**	**3.8 (4.0)**	**10 (11)**	27 (3)
Forward DS (/12)	6.5 (3.1)	8.5 (2.1)	8.4 (3.2)	8.9 (1.8)
Reverse DS (/12)	4.2 (0.9)	7.7 (1.7)	6.0 (3.4)	6.4 (2.1)
GDA (/24)	9.5 (10)	12 (5.8)	11 (7.7)	15 (3.2)
VOSP (/20)	17 (2.5)	**16 (2.4)**	17 (1.3)	18 (2)

Mean (SD) values are shown unless otherwise indicated. Maximum test scores are in parentheses. Scores statistically different from control group performance at p<0.05 are in bold.

*Completed by six participants.

†Including early, prominent anxiety, irritability, paranoia, somatically focussed preoccupations, social phobia (see online supplementary material).

‡Verbal auditory hallucinations.

§Completed by four participants.

¶Data in a separate group of 37 age-matched healthy individuals.

**Significantly superior to both other patient groups.

BPVS, British Picture Vocabulary Scale; C9ORF72, pathogenic expansions associated with C9ORF72; CBI, Cambridge Behavioural Inventory: Wedderburn *et al*^41^; D-KEFS Stroop (response inhibition), Delis-Kaplan Executive Function System; DS, digit span; FTD, frontotemporal dementia; GDA, Graded Difficulty Arithmetic; GNT, Graded Naming Test; MMSE, Mini-Mental State Examination score; MAPT, pathogenic mutations in the microtubule-associated protein tau gene; NA, not applicable; NART, National Adult Reading Test; RMT, Recognition Memory Test; TASIT, The Awareness of Social Inference Test; VOSP, Visual Object and Space Perception battery; WASI, Wechsler Abbreviated Scale of Intelligence.

### Experimental tests

#### General structure

In designing the experimental battery, we set out to sample processes relevant to the perception and cognitive evaluation of body schema and the sense of agency of self versus others acting on that schema. We selected four experimental tasks based on previous neuropsychological evidence demonstrating the usefulness of each task for assessing the relevant body schema process and incorporating simple, uniform response procedures suitable for use in cognitively impaired patients. We assessed perceptual encoding of spatial signals on the body surface using tactile two-point discrimination thresholds^33^; modulation of proprioceptive localisation of limb position using a tendon vibration paradigm^16^ ^20^; body part representation and plasticity using a rubber hand illusion paradigm^30^ ^34^; and explicit attribution of agency in somatosensory signals to self versus others, using a modified version of a previously described tactile stimulation (‘tickle’) paradigm.^19^ ^22^ No feedback was given to participants about their performance during the tests and no time limits were imposed. Further details about the experimental rationale and procedures are in online supplementary material.

#### Tactile two-point discrimination

Tactile two-point discrimination thresholds were determined using an adapted procedure^33^ in which a standard clinical two-point aesthesiometer was applied along the transverse axis of the blindfolded participant's dominant palm; ascending and descending psychophysical series were administered, and a mean two-point discrimination threshold over six series was entered for each participant into group analyses.

#### Proprioceptive localisation under tendon vibration

**Figure 2:**
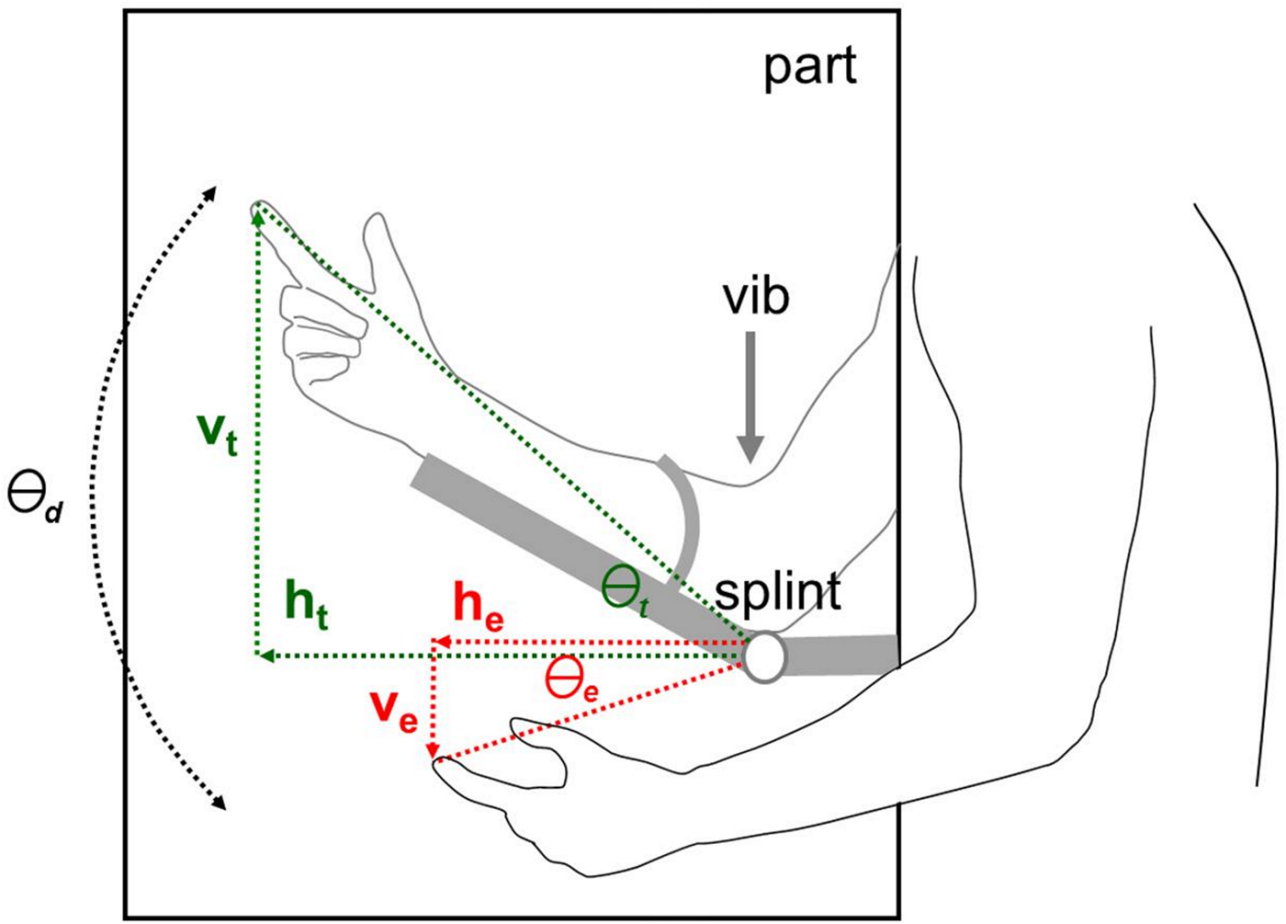
Schematic diagram of the experimental set-up in the proprioceptive localisation task. For clarity, angles have been exaggerated and the fixed (reference, stimulated) arm is shown ‘transparently’ behind the plane of the central partition, **part** on which participant position matching estimates were marked and above the participant's free (localising) arm. The participant's fixed arm was supported by the adjustable **splint** hinged at the elbow; the angle of the splint was varied randomly (either +22.5° or −22.5° relative to horizontal) from trial to trial, and during stimulation trials the vibrator, **vib** was applied to the biceps tendon of this arm. The horizontal (**h_t_**) and vertical (**v_t_**) coordinates of the true position of the target index finger of the fixed arm and the horizontal (**h_e_**) and vertical (**v_e_**) coordinates of the estimated position of the target finger are shown. From these measurements relative to the elbow the true angle of the target finger **Θ_t_** and the position estimation angles **Θ_e_** on each trial were calculated trigonometrically. Angles of deviation from the target angle **Θ_d_** were calculated as the difference between **Θ_t_** and **Θ_e_**., for baseline (no stimulation) trials and stimulation (tendon vibration) trials; the absolute value of each participant's mean **Θ_d_** in each condition was entered into the group analysis.

The procedure adapted for this test^16^ ^18^ is represented schematically in figure 2*.* The seated and blindfolded participant's dominant arm was lightly secured to a hinged splint, and the actual position of the participant's reference index finger was marked on a vertical partition while elbow flexion (at 22.5° or −22.5° relative to horizontal) was passively manipulated by the experimenter. The participant was then asked to oppose the free (non-dominant) index finger as closely as possible to the estimated position of the pointing dominant index finger on the other side of the partition, while randomly ordered flexion angles of 22.5° or −22.5° were applied at the secured elbow (baseline proprioceptive localising accuracy; mean of six trials). This procedure was then repeated while stimulating the biceps tendon of the secured arm at approximately 80 Hz using a customised mechanical vibrator to induce an illusion of elbow extension (10 trials at each flexion angle, randomly ordered; 20 stimulation trials in toto). The position of the participant's proprioceptive matching estimate for each trial was recorded; in off-line analyses, absolute mean deviation angles for each participant's estimates relative to the true target angle (see figure 2) were derived as indices of proprioceptive localisation accuracy in the baseline and stimulation conditions.

#### Rubber hand illusion

**Figure 3:**
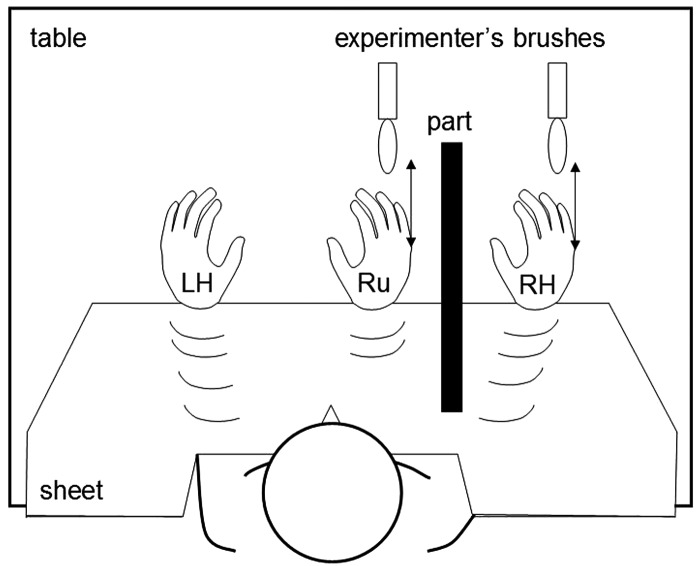
Schematic diagram of the experimental set-up in the rubber hand illusion task. LH, left hand; part, partition; RH, right hand; Ru, rubber hand. See text for further explanation.

The adapted experimental procedure for this test^17^ is shown schematically in figure 3. The participant was seated comfortably at a table wearing rubber gloves; a rubber hand was placed visibly on the table alongside the participant's dominant hand which was obscured by a partition. Both hands were stroked synchronously using a paintbrush for 3 min while the participant watched the rubber hand. The participant then completed a questionnaire (see online supplementary material) to assess the presence and extent of any somatosensory illusion during stimulation: responses were graded using a 7-point Likert scale (1, signifying a strong percept; 7 signifying no percept; highest possible score 21) and each participant's summed score was entered into group analyses.

#### Self versus non-self action attribution

**Figure 4:**
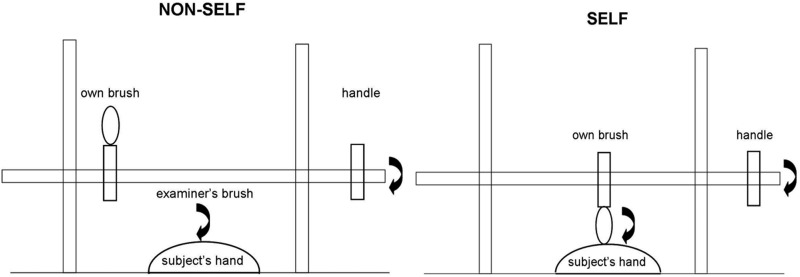
Schematic diagram of the experimental set-up in the ‘self’ versus ‘non-self’ attribution task conditions. See text for further explanation.

The experimental procedure for this test^22^ is represented schematically in figure 4*.* A paintbrush was suspended using a cross-clamp from a rod positioned between two table-mounted retort stands, such that it could be rotated freely by manipulating a handle at one end. The blindfolded participant was positioned with the dominant hand resting palm down on the table between the retort stands, and the apparatus was adjusted so that the paintbrush lightly tracked across the skin of the hand when rotated. On each trial, the handle was rotated by the participant using the non-dominant hand, and the paintbrush was randomly moved along the suspended rod between trials, so that the brush would either contact the participant's hand (‘self’ condition) or would not contact the participant's hand (‘non-self’ trials); on ‘non-self’ trials, the experimenter delivered the tactile stimulus by using an identical paintbrush, either in time with the participant's own handle action (synchronous condition) or with a short delay (around 1 s; asynchronous condition). The task on each trial was to decide whether the tickle stimulus was generated by the participant's own action or by that of the experimenter. Thirty randomly ordered trials were administered (10 self, 10 non-self synchronous, 10 non-self asynchronous), and participant responses were recorded for off-line analysis.

### Statistical analyses

All behavioural data were analysed using Stata12. Data on all behavioural subtests were first assessed to ascertain whether the distribution of scores on that subtest was normal. Where parametric normality assumptions were met, patient groups were compared with the healthy control group, and to each other, using analysis of variance models implementing F tests and two-tailed t tests. Where normality assumptions were not met, Kruskal–Wallis and Mann–Whitney U tests were used. A statistical threshold p<0.05 was taken as the criterion of significance for all tests. As the observations here were made on non-independent behavioural data, corrections for multiple comparisons were not employed, in line with standard statistical practice.^35^

## Results

### Demographic and clinical characteristics

Demographic and clinical characteristics for each of the patient groups and the healthy control group are summarised in table 1. All groups were well matched for age (F_3,26_=0.47, p>0.05), gender (F_3,26_=0.3, p>0.05), handedness (F_3,26_=1.3, p>0.05), and clinical symptom duration was well matched between the patient groups (F_2,14_=1.4, p>0.05).

### General neuropsychological findings

General neuropsychological findings for each of the patient groups and the healthy control group are summarised in table 1. The findings corroborated the syndromic diagnosis of FTD in the patient groups: all three groups performed inferiorly to the healthy control group on standard neuropsychological tests of general intellectual, executive, social cognition, episodic and semantic memory functions, with sparing of short-term memory and posterior cortical functions. Graded naming performance was reduced in the MAPT-FTD group (t_14_=−3.89, p=0.002) and the sporadic-FTD group (t_14_=−2.5, p=0.02) compared with the C9ORF72-FTD group. Patient groups did not differ significantly on any other standard neuropsychological measures: in particular, there were no group differences on IQ, general cognitive capacity (Mini-Mental State Examination), executive, or social-cognition measures. As patient groups were well matched for potentially relevant clinical and neuropsychological characteristics, and as general neuropsychological characteristics were not anticipated a priori to be correlated with performance on any of the experimental tasks, nuisance covariates were not included in analyses of the experimental test data.

### Experimental task performance

Performance profiles for each of the patient groups and the healthy control group on the experimental tasks are summarised in table 2. Mean performance in the C9ORF72-FTD group was significantly inferior to the healthy control group and the other FTD groups on all the experimental measures. Individual data for each test are presented in online supplementary material figure S1; these data generally indicate wide individual variation within disease groups, with overlap between groups.

**Table 2:** Experimental task performance of patient and healthy control groups

	C9ORF72	MAPT	Sporadic-FTD	Healthy controls
Tactile discrimination				
Number completing test	5	6	3	9
Mean (SD) threshold (mm)	18.5 (4.1)*	13.4 (2.6)	10.3 (6.5)	13.1 (3.2)
Range thresholds (mm)	12–23	9–16	4–18	6–18
Tendon vibration				
Number completing test	4	5	4	12
*Baseline accuracy*				
Mean (SD) angle† score (degrees)	4.8 (3.4)	5.8 (5.4)	**6.7 (4.2)**	2.4 (2.0)
Range angle scores (degrees)	0.8–9.2	0.3–13.5	3.1–12.6	0.6–6.3
*Stimulation accuracy*				
Mean (SD) angle score (degrees)	**15.5 (18)**	7.01 (3.2)	4.84 (3.8)	3.8 (3.7)
Range angle scores (degrees)	4–43	4–11	1–9	0–10
Rubber hand illusion				
Number completing test	4	7	4	13
Mean questionnaire score	5 (1.8)*	17.3 (5.2)	16.5.4 (6.8)	13.1 (7.1)
Range questionnaire scores	3–7	9–21	7–21	3–21
Self-non-self action attribution				
Number completing test	5	6	5	13
*Tickle self*				
Mean (SD) score (/10)	7.2 (2.2)*	9.8 (0.4)	10 (0)	9.8 (0.3)
Range scores	5–10	9–10	10	9–10
*Tickle non-self synchronous*				
Mean (SD) score (/10)	4 (0.7)	5.5 (1.5)	5.4 (3.4)	4.6 (3.2)
Range scores	3– 5	3–7	1–10	0–9
*Tickle non-self asynchronous*				
Mean (SD) score (/10)	**6.2 (3.5)**	9.1 (1.2)	8.8 (2.7)	9.9 (0.2)
Range scores	1–9	7–10	10	9–10

Mean (SD) values shown for experimental task performance. Scores statistically different from control group performance at p<0.05 are in bold.

*Also significantly different from both other patient groups; C9ORF72, pathogenic expansions associated with C9ORF72; FTD, frontotemporal dementia; MAPT, pathogenic mutations in the microtubule-associated protein tau gene.

†All angle values are based on individual absolute mean values of deviation angle (see figure 2).

#### Tactile two-point discrimination

Mean tactile two-point discrimination threshold for the C9ORF72-FTD group was significantly higher than for the healthy control group (t_12_=2.54, p=0.02). By contrast, mean thresholds for the MAPT-FTD group and the sporadic-FTD group did not differ significantly from healthy control participants (MAPT-FTD: t_13_=0.18, p>0.05; sporadic-FTD: t_10_=−1.08, p>0.05). Comparing patient groups, the C9ORF72-FTD group had a significantly higher mean tactile discrimination threshold than the MAPT-FTD group (t_13_=2.45, p=0.03) and the sporadic-FTD group (t_10_=2.18, p=0.03).

#### Proprioceptive localisation

Proprioceptive localisation accuracy in the absence of tendon stimulation did not differ between the healthy control group and either of the genetically defined patient groups (C9ORF72-FTD, t_14_ 1.21, p>0.05; MAPT-FTD, t_15_=1.82, p>0.05), though the sporadic-FTD group performed inferiorly to healthy participants in this condition (t_14_=2.11, p=0.05). Under tendon vibration, however, proprioceptive localisation in the C9ORF72-FTD group was significantly less accurate than for healthy participants (t_14_=2.65, p=0.02). By contrast, neither the MAPT-FTD group nor the sporadic-FTD group showed a deficit relative to the healthy control group on this task (MAPT-FTD t_15,_=−0.58, p>0.05; sporadic-FTD, t_14_=0.48, p>0.05). There were no significant differences between the C9ORF72-FTD group and other patient groups on this task.

#### Rubber hand illusion

The mean score relating to the rubber hand illusion questionnaire was significantly different between groups (Kruskal–Wallis χ^2^=8.27, p=0.04). A Mann–Whitney U test revealed that the rubber hand illusory percept was stronger in the C9ORF72-FTD group compared to healthy control participants (p=0.05), while scores for the MAPT-FTD group and the sporadic-FTD group did not differ significantly from healthy participants (both p>0.05). Comparing patient groups, Mann–Whitney rank estimates revealed a significantly greater illusory perceptual effect of the rubber hand in the C9ORF72-FTD group compared to the MAPT-FTD group (p<0.006) and the sporadic-FTD group (p=0.05).

#### Self versus non-self action attribution

A Mann–Whitney U test revealed that differentiation of self-generated from externally generated actions was significantly impaired in the C9ORF72-FTD group relative to the healthy control group in the self-generated (p=0.005) and asynchronous non-self (p=0.0004) tickle conditions. By contrast, neither the MAPT-FTD group nor the sporadic-FTD group showed a deficit for either of these conditions (both p>0.05); nor were there any significant group differences for the synchronous non-self ‘control’ condition (p>0.05). When patient groups were compared, a Mann–Whitney U test further indicated a performance deficit in determining self-generated actions in the C9ORF72-FTD group compared to the other groups (MAPT-FTD, p=0.04; sporadic-FTD, p=0.02); and trends toward inferior performance in the asynchronous non-self tickle condition for the C9ORF72-FTD group compared to the other groups (MAPT-FTD, p=0.07; sporadic-FTD, p=0.06).

## Discussion

Here we have shown using a novel, physiologically motivated paradigm that C9ORF72-FTD is associated with deficits of body schema relative to healthy older individuals. These deficits span levels of body schema processing from tactile encoding (two-point discrimination) and modulation of proprioceptive signals (tendon vibration), through representation of body parts (rubber hand illusion), to cognitive attribution of the agency of somatosensory signals to self versus others (essential for maintaining a stable self-image^25^ ^36^). Our findings further suggest a qualified specificity of these body schema alterations for C9ORF72-FTD versus MAPT-FTD and sporadic FTD. The findings are unlikely to have been attributable to non-specific or confounding effects from general cognitive capacity or disease severity as the FTD groups were well matched for these other characteristics; furthermore, the somatosensory processes implicated are unlikely to have imposed substantial executive or other extraneous task-related cognitive demands. This is in line with previous evidence that body schema processing deficits may develop in C9ORF72-FTD patients with relatively preserved general intellect.^22^ A peripheral sensory basis is similarly unlikely, based on the clinical and electrophysiological findings. We propose altered body schema processing as a plausible, generic pathophysiological mechanism that could potentially underlie various clinical features identified as hallmarks of C9ORF72-FTD in previous work.^3^ ^4^ ^10–12^ While more florid psychotic features (delusions and hallucinations) have been emphasised in C9ORF72-FTD,^4^ the mechanism we propose here is potentially of much wider relevance. It might, for example, contribute to the prominent, otherwise unexplained somatosensory symptoms, social phobias, anxiety, paranoia and other specific interpersonal difficulties as well as the loss of empathy these patients experience^1^ ^21^ ^23^ ^26^; frank psychosis may be a key phenotypic marker within this wider neurobehavioural profile. Neuropsychiatric symptoms are often complex and multidimensional and we do not, of course, argue that altered body schema processing is the sole substrate for such symptoms. Rather, the present findings suggest that neuropsychiatric symptoms may have disease-specific mechanisms within broad syndrome categories such as FTD.

Body schema processing is likely to depend on the integration of multimodal sensory signals and integration of sensory with internal motor efference signals.^19^ ^21^ ^25^ These operations may entail comparison of incoming sensory signals with stored representations and calibration of a prediction error within a feed-forward model in which predictions about the sensory consequences of actions are compared with incoming perceptual information. In this model, parietal cortex and posterior insula play a crucial role in integrating multisensory and sensorimotor representations, and the cerebellum acts as a comparator between incoming afferent signals and outgoing motor commands.^19^ ^30^ ^31^ The interpretation of agency has been shown to recruit a distributed network including thalamus and posterior parietal cortex for transmission and updating of the sensory consequences of actions and prefrontal and cingulate cortex for cognitive appraisal of integrated percepts.^31^ Impaired ability to distinguish the sensory consequences of own from others’ actions, and enhanced bodily illusions shown by our C9ORF72-FTD group might, therefore, be attributable to impaired prediction coding in the cerebellum, or defective integration of sensory percepts by thalamus, parietal or prefrontal cortex or their connections.^19^ ^36^ Although neuroanatomical correlation was not possible here, the elements of this distributed network were previously implicated in neuroimaging^3^ ^6^ ^14^ and neuropathological^3^ ^5^ ^10^ studies of patients with C9ORF72 expansions. Enhancement of the rubber hand illusion in our C9ORF72-FTD group suggests increased plasticity of body part representations, which in turn would be consistent with the finding of impaired self/non-self differentiation on the action attribution task. From a clinical perspective, these findings align C9ORF72-FTD pathophysiologically with schizophrenia, phantom limb phenomena, thalamic strokes and other entities accompanied by abnormal enhancement of body schema plasticity, or a breakdown in the normal boundaries of the schema.^15^ ^21^ ^31^ ^34^ Body schema alterations in our C9ORF72-FTD group also extended to the more elementary processing required to encode somatic spatial relations or postural change, in the two-point discrimination and proprioceptive localisation tasks. Deficits of tactile discrimination have been identified in paradigmatic disorders of body schema, including schizotypy (liability to schizophrenia^33^) and anorexia nervosa,^28^ and are likely to index a fundamental abnormality of low-level somatic coding. The conjunction of impaired tactile spatial acuity and normal baseline proprioceptive acuity in C9ORF72-FTD would be consistent with the existence of multiple neural representations for these functions within the multimodal body matrix.^15^ ^21^

Taken together, our findings suggest that C9ORF72-FTD is associated with loss of body schema definition and abnormally enhanced modulation of body schema boundaries. Involvement of multiple levels of body schema processing is consistent with dysfunction of the common distributed cortico-subcortical network previously identified in this disease. Emerging functional neuroanatomical evidence suggests that the body schema processing hierarchy behaves as a unit, with transformation of information and reciprocal interactions among network elements in health, and under the impact of disease states.^37–39^ From a clinical perspective, body schema alterations associated with C9ORF72-FTD would, in principle, be relatively straightforward to detect and track in individual patients or in the context of clinical trials.

This study has several limitations that suggest directions for future work. Case numbers were small and performance profiles were, in general, variable within disease groups, limiting power to detect effects and precluding direct neuroanatomical correlation. The present findings should, accordingly, be interpreted with caution and await further substantiation. Future work should engage larger (multicentre) patient cohorts, including conditions, such as motor neuron disease, GRN-FTD and the spinocerebellar ataxias that might also be predicted to show deficits of body schema processing on neuroanatomical and neurophysiological grounds. Other dimensions of body schema processing, and the relations between those dimensions and particular neuropsychiatric symptoms, should be explored and correlated with neuroanatomical data. Longitudinal studies will be required to establish whether altered body schema processing is an early hallmark of C9ORF72 mutation carrier status.^33^ Structural and functional neuroanatomical techniques that can capture distributed alterations in network connectivity would allow evaluation of specific hypotheses about the pathophysiology of body schema processing. There is currently considerable interest in understanding how neurodegenerative disease phenotypes and molecular abnormalities map onto brain networks.^40^ Here, we have identified a candidate pathophysiological mechanism of a specific proteinopathy: this mechanism might, in future, yield biomarkers for identifying and tracking C9ORF72-FTD, requiring comparison of body schema metrics with conventional biomarkers across the FTD spectrum. Body schema alterations link more basic self-directed autonomic and homeostatic processes with higher cognition, and we hope that this work will stimulate interest in physiological phenotyping of neurodegenerative diseases more broadly.

## Supplementary Material

Web supplement

Web figure
