# Supplementary material for: Altered body schema processing in frontotemporal dementia with C9ORF72 mutations
Source: J Neurol Neurosurg Psychiatry. 2014 Feb 12;85(9):1016–23. doi: 10.1136/jnnp-2013-306995 (PMC4145454; doi:10.1136/jnnp-2013-306995)
Supplement: Web supplement [file jnnp-2013-306995-s1.pdf]

Altered body schema processing in frontotemporal dementia with C9ORF72 mutations

by LE Downey et al

SUPPLEMENTARY MATERIAL

SUPPLEMENTARY METHODS

Genetic analyses

All patients were screened for pathogenic mutations in genes causing the FTD syndrome, comprising MAPT, GRN, PSEN1, PSEN2 and pathogenic expansions of C9ORF72. If a mutation in the MAPT or GRN gene had been established in the family, this was confirmed in the patient by Sanger sequencing of the specific exon. For the C9ORF72 hexanucleotide repeat, the presence of a large expansion was confirmed by repeat-primed PCR, and subsequently the size was approximated by Southern blot if sufficient DNA was available[S1,S2]. In patients with sporadic disease the absence of a mutation in 17 genes causing dementia was confirmed by targeted next generation sequencing using the MRC dementia gene panel[S1].

Five patients were found to have pathogenic C9ORF72 expansions; of these, three had sufficient DNA for repeat size confirmation (3472, 3501 and 3600 base pairs respectively). A further seven patients had a pathogenic mutation in the MAPT gene (four exon10+16, two exon13 c.1212C>T; one novel presumptively pathogenic mutation c.1052A>G in exon12). All patients with C9ORF72-FTD were known to have had other family members affected with FTD or motor neuron disease, conforming to an autosomal dominant pattern of inheritance. Five patients in the present FTD cohort had no known pathogenic mutation identified, nor any suggestion of a relevant family history, and were therefore classified as having sporadic FTD.

Clinical details

All patients had a typical clinical syndrome of FTD led by behavioural decline and inter-personal difficulties. All patients in the C9ORF72-FTD group exhibited early prominent anxiety, irritability or paranoia; three had somatically focussed preoccupations (obsessive exercising and body image concerns, unexplained somatosensory symptoms, compulsive scratching); and one presented with social phobia. None of the patients with C9ORF72-FTD gave a history to suggest frankly delusional ideation, however one reported auditory hallucinations of voices calling his name. Three patients in the sporadic-FTD group exhibited similar early symptoms, including paranoia, anxiety, somatic preoccupations and agoraphobia; whereas only one patient in the MAPT-FTD group developed an early symptom of this kind (prominent anxiety). On the Cambridge Behavioural Inventory (Wedderburn et al., 2008), the C9ORF72-FTD group showed a similar distribution of total scores to the other FTD groups (Table 1); although unusual beliefs were scored relatively prominently in the C9ORF72-FTD group, these were also reported by caregivers for individual patients in the other FTD groups. None of the patients had symptoms suggesting a major mood disorder at the time of assessment.

Two patients with C9ORF72-FTD had features in keeping with early motor neuron disease (upper limb fasciculations) at the time of their participation; none had typical cerebellar signs or clinical features of peripheral neuropathy. No patients with C9ORF72-FTD had a past history or symptoms suggestive of peripheral neuropathy. Two patients in the sporadic-FTD group had a history of incidental mild peripheral neuropathy (one diabetic and the other of undetermined cause); these patients did not subsequently participate in the tactile discrimination threshold experiment. Clinical electrophysiological studies undertaken in three patients with C9ORF72-FTD corroborated significant denervation in one case; no study showed evidence of peripheral neuropathy. At the time of assessment, two patients in the C9ORF72-FTD group and three patients in the MAPT-FTD

group were prescribed acetylcholinesterase inhibitors; one patient in the C9ORF72-FTD group and one patient in the sporadic-FTD group were prescribed quetiapine.

Structural volumetric brain MRI in 16 patients (contraindicated in one patient with C9ORF72-FTD) corroborated the clinical syndromic diagnosis of FTD. Profiles of brain atrophy were in keeping with those previously described in each FTD syndrome[3,32]: whereas patients with MAPT-FTD showed a comparatively uniform profile of selective atrophy predominantly (and relatively symmetrically) affecting the anterior temporal lobes, atrophy profiles were highly variable in the other disease groups. In particular, regional atrophy profiles exhibited by individuals with C9ORF72-FTD included asymmetric selective frontal atrophy, mild fronto-subcortical atrophy, diffuse atrophy and relatively symmetric mesial temporal lobe atrophy (Figure 1); none of the patients showed definite cerebellar atrophy. No patient had MRI evidence of significant cerebrovascular disease, as assessed by an experienced neuroradiologist.

As part of their clinical evaluation, CSF examination had been undertaken in three patients subsequently shown to have C9ORF72 expansions and in one patient subsequently shown to have a MAPT mutation. In each case, the CSF profile provided no support for underlying concurrent Alzheimer's pathology, with CSF total tau : beta-amyloid$_{1-42}$ ratio <1.

Details of experimental tests

Structure of the experimental test battery

In designing the experimental battery, we set out to sample processes relevant to the perception and cognitive evaluation of body schema and the sense of agency of self versus others acting on that schema. We selected four experimental tasks based on previous neuropsychological evidence demonstrating the utility of each task for assessing the relevant body schema process, where such

evidence was available. In addition, we chose tasks suitable for use in cognitively impaired patients, incorporating simple, uniform response procedures while minimising additional, task-irrelevant cognitive demands (for example, verbal mediation). We assessed perceptual encoding of spatial signals on the body surface using tactile two-point discrimination thresholds (previously identified as a marker for somatosensory dysfunction with schizotypy[33]), measured in a standard psychophysical procedure. Modulation of proprioceptive localisation of limb position was assessed using a tendon vibration paradigm, well documented in the classical neurophysiological literature as an index of proprioceptive acuity and body schema alteration in normal individuals[16,20,S3]: this paradigm capitalises on the propensity of vibration to stimulate muscle spindle afferents and tendon organs[18], thereby producing an illusion of muscle stretch and limb motion at the joint. Body part representation and plasticity were assessed using a rubber hand paradigm: this has previously shown to modulate powerfully body schema boundaries and content in normal individuals, with illusory incorporation of the rubber hand into the body schema[30,34]. The rubber hand illusion is likely to arise at the interface between integration of (tactile and visual) sensory signals and top-down influences that interpret the origins of sensory signals and actions and attribute agency. Finally, we assessed the explicit attribution of agency in somatosensory signals to self versus others, using a modified version of a previously described tactile stimulation ('tickle') paradigm[19,22]: in the healthy brain, this paradigm has been shown to engage a large-scale brain network including the cerebellum.

All experimental test sessions were administered by a single experimenter (LED) to reduce variation in stimulation and assessment criteria. No feedback was given to participants about their performance during the tests and no time limits were imposed on participant responses.

Tactile two-point discrimination

The experimental procedure used for this test was adapted from a previously described procedure[33]. Tactile two-point discrimination thresholds were determined using a standard clinical two-point aesthesiometer lightly applied along the transverse axis of each participant's dominant palm. Prior to commencing the test, it was established that each participant could easily detect the touch of the aesthesiometer. During the test, the participant was seated comfortably and blindfolded, and the task on each trial was to indicate whether one or two points (applied simultaneously) had been detected. Both ascending and descending series were administered, as these have equal validity for threshold determination[S4]. In a descending series, the distance between the two points was incrementally reduced in 2 mm steps from an initial separation of 40 mm, until the participant indicated that 'one point' was detected on two consecutive trials; the first of these successive 'one point' responses was taken as the two point detection threshold for that descending series. In an ascending series, the distance between the two points was incrementally increased in 2 mm steps from an initial separation of 2 mm until the participant indicated that two points were detected on two consecutive trials; the first of the successive 'two points' responses was taken as the two-point detection threshold for that ascending series. Descending and ascending series were each repeated three times, yielding a total of six threshold estimates for each participant; a mean two-point discrimination threshold was calculated by averaging the threshold scores across all six series, and these individual participant mean two-point thresholds were incorporated in subsequent analyses of group tactile threshold differences.

Proprioceptive localisation under tendon vibration

The experimental procedure for this test was adapted from a previously described paradigm (Goodwin et al., 1972; Naito et al., 1999), the procedure is represented schematically in Figure 2. Participants were seated comfortably and blindfolded, with their arms flexed forward at the elbows and separated by a removable vertical cardboard partition. The participant's dominant arm

was lightly secured to a hinged splint such that the angle of elbow flexion could be manipulated passively by the experimenter (the participant was instructed not to attempt actively to move the elbow). The participant touched the cardboard partition with their outstretched dominant index finger while flexion angles of 22.5 ° and -22.5° relative to the horizontal (determined using a protractor) were applied to the secured elbow, and the actual position of this reference finger was marked on the cardboard for each flexion angle, for offline analysis. The participant was then asked to make a pointing gesture with the dominant index finger (no longer in contact with the cardboard) while flexion angles of 22.5 ° and -22.5° were randomly applied at the secured elbow; at each flexion angle, the participant was asked to oppose the free (non-dominant) index finger as closely as possible to the estimated position of the pointing dominant index on the other side of the partition, in order to estimate the baseline accuracy of proprioceptive localisation matching in the absence of any tendon stimulation. Three baseline position matching estimates were marked on the cardboard for each flexion angle, for offline analysis. In the subsequent stimulation trials, this procedure was repeated after biceps tendon stimulation at approximately 80Hz using a customised mechanical vibrator: vibration was applied for 30 seconds to the biceps tendon of the secured arm and continued to be applied as the participant performed the localising task. A total of 20 stimulation trials were administered, comprising 10 trials at each flexion angle, randomly ordered. The position of the participant's proprioceptive matching estimate for each trial was marked on the cardboard for offline analysis.

In the offline analysis, deviation angles for the participant's estimates relative to the true target angle were derived as indices of proprioceptive localisation accuracy in each of the conditions. We used angles rather than absolute position values as angle is intrinsically normalised for individual variations in arm length and is likely to be more relevant to the physiological parameter (joint angle) that is controlled by the proprioceptive servo mechanism (Goodwin et al., 1972). For each

flexion angle, the actual angle of the participant’s fixed dominant index relative to the elbow was derived using the formula:

true angle = 1/tan (vertical measured index position / horizontal measured index position)

The angles of the participant’s position estimates at baseline and under tendon vibration were derived using the formula:

estimation angle = 1/tan (vertical measured position estimate / horizontal measured position estimate)

These values were converted to degrees using an arctan function and the angles by which the participant’s estimates deviated from the true angle at baseline and under tendon stimulation were calculated as:

deviation angle = estimation angle - true angle

Deviation angle values were averaged in order to generate a mean deviation angle at baseline (indexing the baseline accuracy of proprioceptive localisation) and a mean deviation angle under tendon stimulation (indexing the perceptual effect of tendon vibration) for each flexion angle, for each participant. Absolute values of these individual mean deviation angles were entered in subsequent group analyses.

Rubber hand illusion

The experimental procedure for this test was adapted from a previously described paradigm (Botvinick & Cohen, 1998), represented schematically in Figure 3. Participants were seated comfortably at a table wearing rubber gloves with both hands facing forward palms-down on the table. A vertical partition completely obscured the participant's view of their own dominant hand, while a rubber hand was placed visibly on the table alongside the participant's obscured dominant hand. The rubber hand closely resembled the participant's own gloved hand (to reduce potentially confounding visual or auditory cues during the stimulation procedure) and the origins of the participant's own hands and the rubber hand were obscured by a sheet in order further to enhance the illusion that the rubber hand was the participant's own dominant hand. Light tactile stimulation was delivered using a paintbrush (14.5 × 1 cm, 1"25 bristles) on the index finger of the participant's dominant hand and synchronously simulated in identical fashion on the rubber hand for three minutes. The participant's task during stimulation was to watch the brush stroking the hand in front of them. On cessation of stimulation, the participant completed a questionnaire (administered verbally by the experimenter) to assess the presence and extent of any somatosensory illusory experience during stimulation. This questionnaire (adapted from Botvinick and Cohen, 1998; see Supplementary Material on-line) comprised target items (for example, 'Did you feel as if the rubber hand was your own hand?') interspersed with foil items (for example, 'Did you feel your real hand turning rubbery?') and responses were graded using a 7-point Likert scale, a score of 1 signifying a strong percept and a score of 7 signifying no percept. Each participant's scores on the Likert scale were summed for the three target items, such that a higher score indicated a stronger illusory percept (highest possible score = 21). These summed scores were entered for each participant in subsequent group analyses.

Self versus non-self action attribution

The experimental procedure for this test has been described previously (Downey et al., 2012); the procedure is represented schematically in Figure 4. A paintbrush (14.5 × 1 cm, 1”25 bristles) was suspended by using a cross-clamp from a rod positioned between two table-mounted retort stands, such that the rod (and the attached paintbrush) could be rotated freely by manipulating a handle attached to one end. The participant was positioned with the dominant hand resting palm-down on the table between the retort stands, and the apparatus was adjusted so that the paintbrush lightly tracked across the skin of the hand when the handle was rotated by the subject, using the non-dominant hand. During the experiment, the paintbrush was randomly moved along the suspending rod from trial to trial, such that the brush either would contact the participant’s hand (“self” condition) or would not contact the participant’s hand (“non-self” trials); on “non-self” trials, the experimenter delivered the tactile stimulus by using an identical paintbrush, either in time with the participant’s own action (synchronous condition) or with a short delay (around 1 second; asynchronous condition); the synchronous condition here was intended as a control condition. The retort-mounted paintbrush was shifted by the experimenter before every trial (whether self or non-self) to minimize any extraneous cues from sound or the absolute position of the brush. Participants were blindfolded and instructed to rotate the handle three times in every trial: the task on each trial was to decide whether the tickle stimulus was generated by the participant’s own action or by that of the experimenter. It was established before commencing the experiment that participants were able reliably to detect the sensory stimulus delivered by the brush. Thirty experimental trials were administered, comprising 10 self, 10 non-self synchronous, and 10 non-self asynchronous trials in randomised order. Participant responses were recorded and stored for offline analysis.

SUPPLEMENTARY RESULTS AND DISCUSSION

Individual participant data

Individual data for each test are plotted in Figure S1; of note, the two patients with clinical features of early motor neuron disease performed within the overall C9ORF72-FTD group range across the experimental tests, indicating that these patients' mild motor features were not driving the group profile. Individual mean tactile discrimination thresholds fell above the healthy control range (and indeed, above the MAPT-FTD and sporadic-FTD ranges) for most patients with C9ORF72-FTD. The magnitude of individual proprioceptive errors under tendon vibration varied widely among individual patients with C9ORF72-FTD: indeed, the group difference relative to healthy controls on this test was attributable to one patient with C9ORF72-FTD who experienced a very strong illusion of elbow extension. This patient had a history of unexplained somatosensory symptoms, anxiety and paranoia but showed normal baseline proprioceptive acuity relative to the healthy control group. Individual rubber hand questionnaire scores were more tightly clustered for patients with C9ORF72-FTD than in the other experimental groups, suggesting that this enhanced perceptual illusion was a relatively consistent finding within the C9ORF72-FTD group. Individual scores for the asynchronous non-self tickle condition fell below the healthy control range for most patients with C9ORF72-FTD.

The question arises whether abnormalities of peripheral sensory pathways might account for increased tactile threshold in patients with C9ORF72-FTD. This is unlikely: no patients included in the present C9ORF72-FTD cohort had clinical or electrophysiological evidence of peripheral sensory dysfunction, nor is it likely that this finding is related simply to abnormal efferent influences on sensory traffic associated with motor neuron disease, as only two of the patients had mild clinical signs of amyotrophy. Some caution is required in interpreting the specificity of the tactile discrimination deficit as the disease group comparisons did not reach the statistical criterion for a significant inter-group difference, likely due to the small case numbers. A similar caveat applies to the findings on the proprioceptive localisation task, as only one patient with

C9ORF72-FTD demonstrated substantially reduced proprioceptive accuracy under tendon vibration, albeit in the direction predicted with an enhanced illusion of limb stretch. This effect was a true index of illusory modulation of a specific postural cue contributing to body schema, since baseline proprioceptive accuracy in this case was normal. Neither the healthy control group nor the patient groups showed a consistent direction of perceptual modulation on the proprioceptive matching task: the basis for this is uncertain, though it has been shown previously that healthy older individuals are less efficient and less accurate in explicit proprioceptive localisation tasks and under perceptual illusions than their younger counterparts[S5].

SUPPLEMENTARY REFERENCES

S1. Beck J, Pittman A, Adamson G, Campbell T, Kenny J, Houlden H et al. Validation of next generation sequencing technologies in genetic diagnosis of dementia. *Neurobiol Aging* 2013, in press.

S2. Renton AE, Majounie E, Waite A, Simón-Sánchez J, Rollinson S, Gibbs JR, et al. A hexanucleotide repeat expansion in C9ORF72 is the cause of chromosome 9p21-linked ALS-FTD. *Neuron* 2011;72:257-268.

S3. Lackner JR. Some proprioceptive influences on the perceptual representation of body shape and orientation. *Brain* 1988;111:281-297.

S4. Kolb B, Wishaw IQ. *Fundamentals of Human Neuropsychology*, 6th Ed. Worth: Richmond, 1996.

S5. Boisgontier MP, Nougier V. Ageing of internal models: from a continuous to an intermittent proprioceptive control of movement. *Age* 2013; 35: 1339-1355.

Figure S1

Scatter plots of individual data for experimental tests. Note change of vertical scale between plots. 2PD, two-point tactile discrimination threshold (millimetres); Asynch, asynchronous stimulation condition (see text); C9ORF72, pathogenic expansions associated with C9ORF72; HC, healthy control individuals; MAPT, pathogenic mutations in the microtubule-associated protein tau gene; Proprioceptive error, raw mean angle (degrees) of deviation of matching estimate from target under biceps tendon stimulation, where negative values correspond to perceived increasing elbow extension (see text); Rubber hand illusion, rating of intensity of percept on Botvinick and Cohen questionnaire (see below); spFTD, sporadic frontotemporal dementia.

The rubber hand questionnaire (after Botvinick and Cohen[17])

<u>It seemed as if I were feeling the touch of the paintbrush in the location where I saw the rubber hand touched</u>

1	2	3	4	5	6	7
**Strongly Agree**						**Strongly Disagree**

<u>It seemed as though the touch I felt was caused by the paintbrush touching the rubber hand</u>

1	2	3	4	5	6	7
**Strongly Agree**						**Strongly Disagree**

<u>I felt as if the rubber hand were my hand</u>

1	2	3	4	5	6	7
**Strongly Agree**						**Strongly Disagree**

<u>It felt as if my (real) hand were drifting towards the right (towards the rubber hand)</u>

1	2	3	4	5	6	7
**Strongly Agree**						**Strongly Disagree**

<u>It seemed as if I might have more than one left hand or arm</u>

1	2	3	4	5	6	7
**Strongly Agree**						**Strongly Disagree**

<u>It seemed as if the touch I was feeling came from somewhere between my own hand and the rubber hand</u>

1	2	3	4	5	6	7
**Strongly Agree**						**Strongly Disagree**

<u>It felt as if my (real) hand were turning ‘rubbery’</u>

1	2	3	4	5	6	7
**Strongly Agree**						**Strongly Disagree**

<u>It appeared as if the rubber hand were drifting towards my hand</u>

1	2	3	4	5	6	7
**Strongly Agree**						**Strongly Disagree**

<u>The rubber hand began to resemble my own (real) hand</u>

1	2	3	4	5	6	7
**Strongly Agree**						**Strongly Disagree**

The first three questions relating to the illusory percept were scored here; the remaining questions relate to suggestibility.
