# Supplementary figures and images for: Altered body schema processing in frontotemporal dementia with C9ORF72 mutations
Source: J Neurol Neurosurg Psychiatry. 2014 Feb 12;85(9):1016–23. doi: 10.1136/jnnp-2013-306995 (PMC4145454; doi:10.1136/jnnp-2013-306995)

## Supplementary Figure

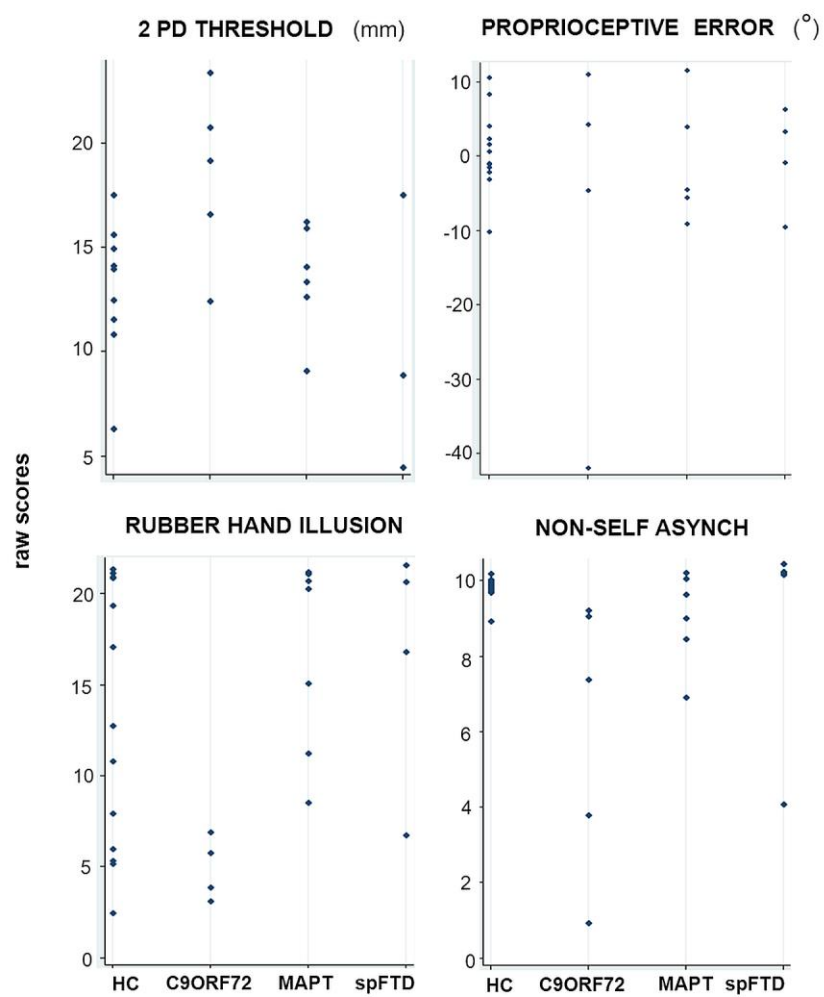

Supplement: Web figure [file jnnp-2013-306995-s2.pdf]
